# Supplementary material for: Spatial Variation in Stream-Water Bacterial Communities Across Sites Differing in Wild Chinese Giant Salamander Detection Frequency
Source: Biology (Basel). 2026 Sep 18;15(18):1647. doi: 10.3390/biology15181647 (PMC13604818; doi:10.3390/biology15181647)
Supplement: Supplementary file 1 [file biology-15-01647-s001.zip › Data Related to “Spatial Variation in Stream-Water Bacterial Communities across Sites Differing in Wild Chinese Giant Salamander Detection Frequency”/Microbial Data/report/src/pictures/picrust2.KO.heatmap.pdf]

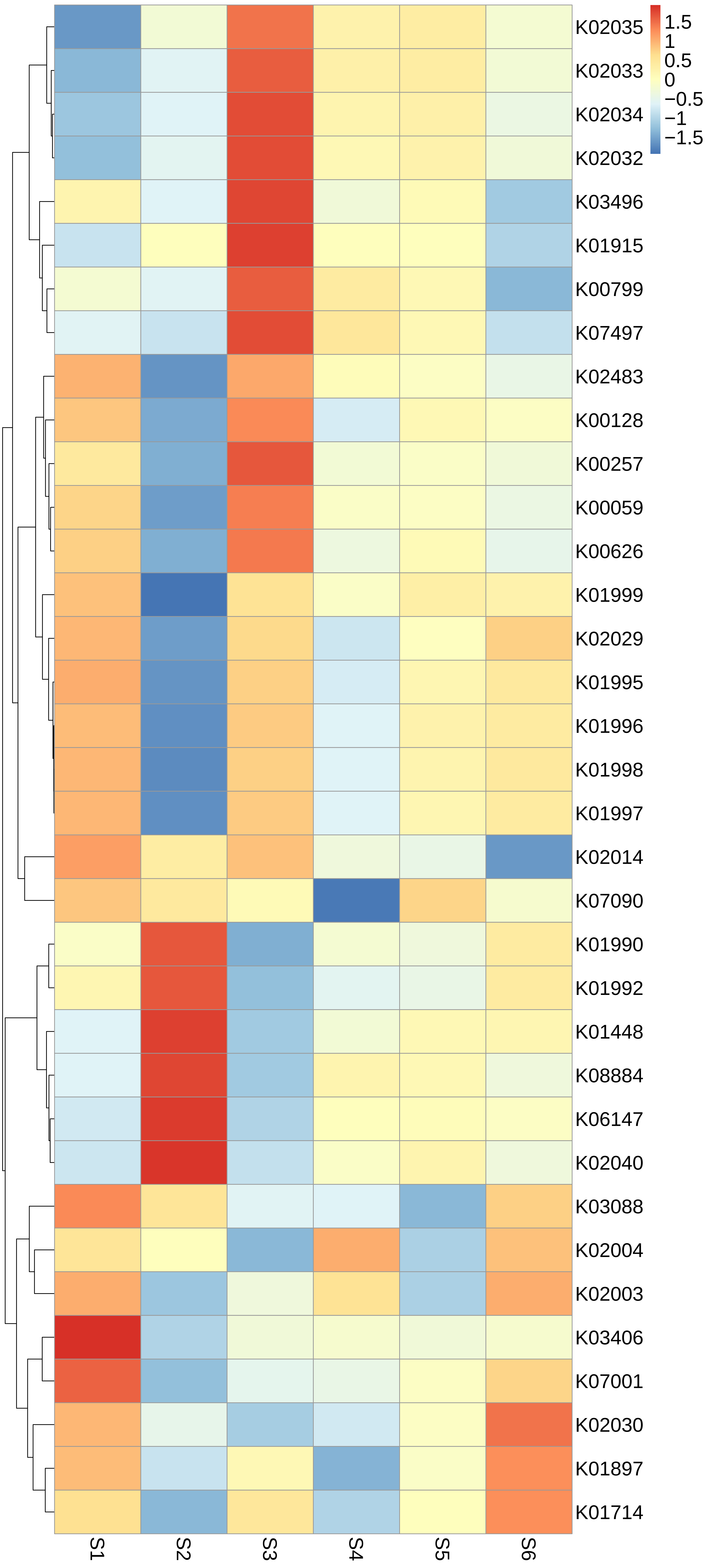

K02035
K02033
K02034
K02032
K03496
K01915
K00799
K07497
K02483
K00128
K00257
K00059
K00626
K01999
K02029
K01995
K01996
K01998
K01997
K02014
K07090
K01990
K01992
K01448
K08884
K06147
K02040
K03088
K02004
K02003
K03406
K07001
K02030
K01897
K01714
S1
S2
S3
S4
S5
S6
1.5
1
0.5
0
−0.5
−1
−1.5
